# Bristling with potential: evaluating the effects of awns on yield under heat stress

**DOI:** 10.1093/jxb/erad392

**Published:** 2023-11-21

**Authors:** Nikolai M Adamski

**Affiliations:** John Innes Centre, Norwich Research Park, Norwich, NR4 7UH, United Kingdom

**Keywords:** Awns, wheat, yield, stress

## Abstract

This article comments on:

**DeWitt N, Lyerly J, Guedira M, Holland JB, Murphy JP, Ward BP, Boyles RE, Mergoum M, Babar MA, Shakiba E, Sutton R, Ibrahim A, Tiwari V, Santantonio N, Van Sanford DA, Howell K, Smith JH, Harrison SA, Brown-Guedira G**. 2023. Bearded or smooth? Awns improve yield when wheat experiences heat stress during grain fill in the southeastern United States. Journal of Experimental Botany **74**, 6749–6759.

**Awns are bristle-like structures present in many grass species. While researchers have identified ecological roles for these organs in natural grass populations, their ‘usefulness’ in grass crop species, such as wheat, has been less clear. DeWitt *et al.* (2023) have leveraged historic data from breeding programmes across the southeastern USA to provide more insights into this question. Their meta-analysis confirms that awned wheat performs better in heat-stressed environments than awnless wheat. Crucially, though, they show that awned wheat is predicted to provide more year-to-year stability in yield, suggesting that awns could be an important trait for adapting crops to changing climatic conditions.**

Watching the wind ripple through a field of barley is a serene and calming experience: the plants bending back and forth, light and shadows dancing across the spikes and the bristle-like awns attached to them. Among the *Poaceae*, the grass family, awns have been gained and lost throughout the course of evolution (Petersen and Kellogg, 2022). The flowers of wheat, just like those of barley, rye, and oat, carry awns. The small cereal flower (commonly called a floret) does not sport bright, beautifully coloured petals; instead, the reproductive organs (three anthers and a single ovary) are enclosed by two leaf-like bracts, the lemma and the smaller palea. Depending on the species, one or more florets form a spikelet, which acts as the seed dispersal unit of wild cereals. The spikelet is subtended by two bracts, called glumes (Fig. 1). Most commonly, awns grow out from the lemma, although some wheat subspecies also carry awns on their glumes (Haque *et al.,* 2011).

**Fig. 1.**
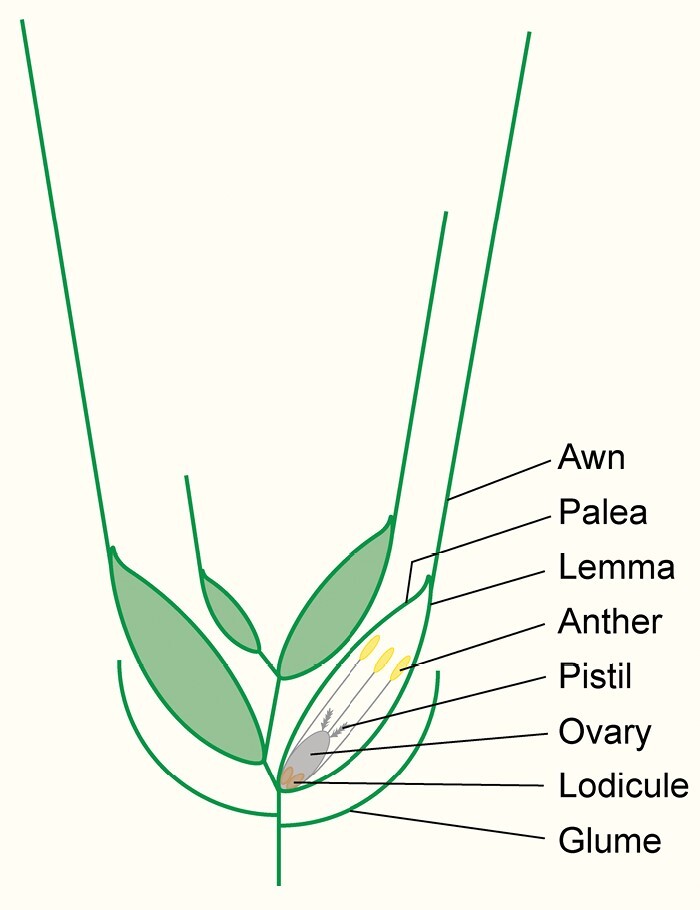
Schematic representation of a wheat spikelet. The image represents a transverse section through a wheat spikelet consisting of four florets, which are enclosed by two glumes. For clarity, the glumes are shown as simple lines. Each floret consists of two leaf-like organs, the lemma and palea, which enclose the reproductive organs (ovary, pistil, and anthers). The floret also contains two lodicules, which swell up at anthesis to push the lemma and palea apart. This allows the anthers to emerge and also enables pollen from other spikelets or plants to fertilize the ovary. The awn is an extension of the lemma mid-vein. Awn length varies both with spikelet position across the wheat spike and with floret position on individual spikelets.

Awns are a vascularized extension of the mid-vein of the lemma/glume; they can be barbed to different degrees and taper into a sharp point. For wild wheat populations, awns are a crucial part of the seed dispersal unit. In a process called epizoochory, the long, barbed awns can attach to animals, which then disperse the spikelets and grains across a wide area. More importantly, the awns are hygroscopic and allow spikelets to move across and burrow into the ground, again assisting with seed dispersal (Elbaum *et al.,* 2007). For cereal crops, however, seed dispersal is irrelevant and counterproductive for agronomic purposes. Although awnless wheat varieties were created during domestication, awns were not eliminated completely during this process; whether this was a result of a lack in diversity, pure aesthetics, or beneficial agronomic properties of awns in at least some environments has been intensely debated.

Many studies have investigated the effects of awns on agronomic performance, especially yield, in near-isogenic sister lines in different environments (Rebetzke *et al.,* 2016; DeWitt *et al.,* 2020; Sanchez-Bragado *et al.,* 2020, 2023). In general, awned wheat varieties produce fewer but larger grains than their awnless sister lines. The result of these studies, on average, is a net-zero effect on grain yield, which led Sanchez-Bragado *et al.* (2023) to ask: ‘Awned versus awnless wheat spikes: does it matter?’.

## New insights into an old question

In the present issue, DeWitt *et al.* (2023) have analysed 12 years’ worth of data from public wheat-breeding programmes in the southeastern USA. The breeding trials were not specifically set up to test the agronomic effects of awns but simply represented efforts to develop better and higher-yielding varieties. The allele status of a single locus (called *B1*) has been shown to be largely predictive for the presence or absence of awns in US hexaploid germplasm (DeWitt *et al.,* 2020). In this manner, DeWitt *et al.* (2023) could retrospectively determine awn status within the breeding trials. With roughly 50% of the entries being awned, this dataset represented a great case study to correlate agronomic traits with environmental factors.

Previous reports have suggested that awned wheat outperforms awnless wheat in terms of yield in hot or dry environments. To test this hypothesis, DeWitt *et al.* (2023) used a mixed linear model to correlate the effects of temperature and precipitation patterns with final yield after correcting for population structure. Specifically, the authors looked at these interactions during the grain-filling period, which is the time following anthesis when the wheat plant shuttles nutrients into its developing grains. No correlation could be detected between yield and precipitation during grain filling. Maximum daytime temperatures during grain filling, however, showed a clear correlation with final yield; both awned and awnless wheat showed a reduction in yield when exposed to high temperatures, but the yield penalty was smaller in the awned lines. On the other hand, the awnless lines outyielded awned varieties in cooler climates.

Looking more closely at the data, the authors concluded that the effect of awns on yield can be highly variable between test sites, years, and germplasm tested. When looking at the variance of these parameters, though, DeWitt *et al.* (2023) noticed that the predicted yield variance for awned lines is generally lower than for awnless germplasm, even in some site–year combinations where awns are negatively correlated with yield. This implies that, although awnless varieties have a generally higher yield potential, the awned lines provide a higher yield baseline. The authors conclude that, coupled with their higher resilience against heat stress, awns may provide greater yield stability, especially when crops face less favourable environmental conditions. This is an incredibly powerful conclusion that could not have been drawn using the small-scale studies typical of most academic research. It also highlights the importance of publicly available data to perform this type of large-scale meta-analysis.

Coming back to the question of whether awns in wheat matter, the answer seems to be one step closer to ‘yes’ than before. From a breeder’s perspective, it largely depends on the type of environment that is targeted. The higher yield potential of awnless wheat has been shown time and again. In areas prone to late-season stress, breeding for heading date could help to evade these stresses while maintaining a high yield potential. Global temperatures are predicted to increase, though, while weather patterns will generally become more variable (IPCC, 2022). The increased yield stability provided by awns could thus be an important factor to select for.

## Implications

From a researcher’s perspective, DeWitt *et al.* (2023) opens up many new questions. The awn status in the tested germplasm was determined by the *Tipped 1* (*B1*) locus. *B1* encodes a C2H2-type transcription factor and its gene expression levels determine awn length, with awnless wheat showing high expression whereas awned wheat has no expression or carries non-functional alleles (DeWitt *et al.,* 2020; Huang *et al.,* 2020; Ke *et al.,* 2023; Li *et al.,* 2023). Would it be possible to fine-tune the expression of *B1* to give the best of both worlds, that is, a high and stable yield potential? In addition to *B1*, two other loci have been described to determine awn status, namely *Tipped 2* (*B2*) and *Hooded* (*Hd*). Is it possible that *B1* has some adverse pleiotropic effects, resulting in higher yield variance and susceptibility to stress? Could these possible pleiotropic effects be avoided in awnless lines carrying *B2* or *Hd* alleles?

The awns within the germplasm analysed in DeWitt *et al.* (2023) were extensions of the lemma. Persian wheat (*Triticum carthlicum*), a tetraploid wheat subspecies, has been reported to carry awns on both lemma and glume. Would wheat germplasm with more awns show even stronger effects in terms of yield stability and resistance to heat stress? Genetic mapping suggests that the causal locus for glume awns is distinct from *B1*, opening up the possibility to separate the two traits (Haque *et al.,* 2011). Would glume awns have fewer negative effects on the yield potential compared with lemma awns?

Another question is that of the physiological reasons for the effects observed by DeWitt *et al.* (2023). Awns have been shown to have a higher transpiration ratio (photosynthesis/transpiration) than flag leaves, with an optimal carbon exchange rate at temperatures exceeding 30 °C (Blum, 1985, 1986). These attributes would suggest that awns, despite their initial cost, would be strong sources for assimilates, which could be easily transported to the nearby developing grains and thus boost grain yield, especially under water-limited conditions. However, grains seem to be equally sink-limited in both awned and awnless wheat lines, arguing against this hypothesis (Sanchez-Bragado *et al.,* 2020).

As mentioned above, awned wheat lines tend to produce fewer but larger grains compared with awnless varieties. A common hypothesis for this effect is the diversion of resources from floret development into awn production, which causes fewer florets to be formed in awned wheat. Could there be a different explanation for the reduction in grain number in awned wheat?

Recently, Backhaus *et al.* (2023) have shown that basal and apical spikelets of awnless wheat form fewer grains than central spikelets—not because of a lack of resources but due to delays in floret development. They have demonstrated that, unlike the availability of assimilate, the developmental age of florets at ~20 days prior to anthesis was highly correlated with the final number of living florets at anthesis. It is important to note that each spikelet initiates a large number of florets (10–12), which are mostly aborted prior to anthesis; a typical wheat spikelet carries between three and five florets at anthesis (Guo *et al.,* 2016). Backhaus *et al.* (2023) have consistently observed that florets in basal and apical spikelets tended to be of a lower developmental age than corresponding florets from central spikelets, suggesting that developmental age was the main reason for their abortion.

Could the outgrowth of awns delay floret development enough that more florets fail to reach a certain threshold age and thus are aborted pre-anthesis? If so, could we modify these developmental behaviours to obtain awned wheats with higher floret numbers and thus higher yield potential?

Many more questions remain to be answered, which is a clear sign that DeWitt *et al.* (2023) have touched upon an important aspect of plant biology and breeding. While the role of awns for wheat is still not fully understood, there is a lot of potential in further studying awns from different research angles.
